# Is the Spatial Distribution of Mankind's Most Basic Economic Traits Determined by Climate and Soil Alone?

**DOI:** 10.1371/journal.pone.0010416

**Published:** 2010-05-05

**Authors:** Jan Beck, Andrea Sieber

**Affiliations:** Department of Environmental Sciences, University of Basel, Basel, Switzerland; University of Arizona, United States of America

## Abstract

**Background:**

Several authors, most prominently Jared Diamond (1997, *Guns*, *Germs and Steel*), have investigated biogeographic determinants of human history and civilization. The timing of the transition to an agricultural lifestyle, associated with steep population growth and consequent societal change, has been suggested to be affected by the availability of suitable organisms for domestication. These factors were shown to quantitatively explain some of the current global inequalities of economy and political power. Here, we advance this approach one step further by looking at climate and soil as sole determining factors.

**Methodology/Principal Findings:**

As a simplistic ‘null model’, we assume that only climate and soil conditions affect the suitability of four basic landuse types – agriculture, sedentary animal husbandry, nomadic pastoralism and hunting-and-gathering. Using ecological niche modelling (ENM), we derive spatial predictions of the suitability for these four landuse traits and apply these to the Old World and Australia. We explore two aspects of the properties of these predictions, conflict potential and population density. In a calculation of overlap of landuse suitability, we map regions of potential conflict between landuse types. Results are congruent with a number of real, present or historical, regions of conflict between ethnic groups associated with different landuse traditions. Furthermore, we found that our model of agricultural suitability explains a considerable portion of population density variability. We mapped residuals from this correlation, finding geographically highly structured deviations that invite further investigation. We also found that ENM of agricultural suitability correlates with a metric of local wealth generation (Gross Domestic Product, Purchasing Power Parity).

**Conclusions/Significance:**

From simplified assumptions on the links between climate, soil and landuse we are able to provide good predictions on complex features of human geography. The spatial distribution of deviations from ENM predictions identifies those regions requiring further investigation of potential explanations. Our findings and methodological approaches may be of applied interest, e.g., in the context of climate change.

## Introduction

Differences in economic traits and the distribution of wealth amongst the peoples of the world are of concern for understanding human history [1] as well as in applied macroeconomics [e.g., [2]–[4]]. Food production is the most basic aspect of economy, as pointed out e.g. in [5]. It is the foundation of sustainable wealth and persistence of societies. Only very few societies, if any, could make themselves independent from local food production for any long periods of time. Four basic types of landuse can be distinguished: Hunting-and-gathering, which directly exploits resources of the land; nomadic pastoralism, which uses domesticated animals to make use of natural primary productivity; agriculture, which controls primary productivity by growing domesticated plants; and sedentary animal husbandry, which uses domesticated animals as addition or alternative to agriculture, making use of primary productivity and/or agricultural products as food for livestock.

While earlier speculations implied, directly or indirectly, that economic differences among the peoples of the world are due to differences between cultures, races or individual governmental decisions, several authors [e.g. 1], [6] have begun to propose a biogeographic explanation of the ultimate reasons behind these differences. According to Diamond's narrative [1], several aspects of ecological and geographic conditions, such as the availability of plants and animals suitable for domestication, were pivotal to the different timings of the transition from hunter-and-gatherer lifestyles to agriculturalist or pastoralist societies (i.e., the ‘neolithic revolution’, [7]). Resulting changes in population densities then had cascading effects of societal and cultural development. While Diamond's supporting evidence [1] was largely anecdotal, subsequent statistical modelling and testing supported the validity of the general idea [e.g., [8]–[10], but [see 11]].

Diamond [1] also provided manifold examples of how consequent advantages lead to a recurring pattern of cultural or physical genocide of hunter-and-gatherers by agriculturalists throughout history [see also 12], [13]. Reports of conflicts between followers of different landuse forms are known since the earliest of written history (e.g. agriculturalist *vs.* nomads, cf. Cain *vs.* Abel), and they can still be seen at least as indirect factors in some modern-day conflicts.

In this paper we explore the hypothesis that the spatial distribution of basic landuse types is determined solely by climate and soil conditions. This can be viewed as a ‘null model’ [14] for historical and social research on the topic: What would we expect if there were no historical legacies, no cultural preferences or other contingencies, but if all people had chosen their landuse in light of the suitability of local climate and soil? In particular, we are interested in what regions large deviations occur between our model and reality. Here, we propose, further investigation on additional, e.g. cultural and historical factors may be particularly worthwhile.

To map the spatial distribution of the suitability for landuse types we used ecological niche modelling (ENM), an approach commonly applied to estimate geographic distributions of species [15] (i.e., we replaced ‘species’ with ‘landuse type’). We are not aware of previous attempts to predict cultural traits with this method, but there are other exemplar cases of concepts and models from ecology and evolution proving successful to research questions in cultural systems [16]–[18].

Specifically, we explored the output of our ‘null model’ predictions for the following questions.

1. How good do the models match reality, and where do they differ?
2. In what regions are there conditions of similar quality for different landuse types? Actual landuse in these regions will be ambivalent to predict, but these regions may highlight potential conflict zones between peoples of different landuse traditions.
3. How are population densities and economic “power” related to modelled suitability of landscapes for agriculture, and where are deviations?

## Methods

We restricted the geographic extent of our research to the Old World including Australia (i.e., 25°W–180°E longitude, 60°S–90°N latitude). We worked in a spatial resolution of ca. 5×5 km raster cells (i.e., grid cells with a side length of 2.5 arc minutes, working in geographical projection). All necessary manipulation of data as well as map creation was carried out in a geographic information system (GIS; software ArcGIS 9.2).

### Economic trait data

For each of the four landuse types (hunting-and-gathering, nomadic pastoralism, sedentary animal husbandry and agriculture) we compiled between 47 and 290 ‘presence records’, respectively. Each one indicated a site (defined by geographical coordinates) where this form of landuse is currently practised. To reduce data non-independence due to spatial autocorrelation [19] and cultural contingencies, we used only few site records within each ethnic group, defined by cultural and linguistic categories. This should reduce the effect of members of ethnic groups sharing, among many other cultural traditions, their ways of landuse. See Table S1 for details, data and sources.

### Environmental data

We used climate data from [20], a compilation of monthly data of 30 year averages (1961–1991) that were interpolated to almost the entire world. These data entered the model as continuous data, whereas a soil classification from the Food and Agriculture Organization of the United Nations (FAO, World Soil Resources Coverage; http://www.fao.org/ag/agll/wrb/soilres.stm) was rasterized and used as categorical data. Data were reclassified to the latest FAO soil type classification (32 soil types).

### Modelling and model evaluation

Ecological niche modelling has been championed in recent years to provide good estimates of species' geographic ranges despite limited survey data being available [15], [21], [22]. We used maximum entropy modelling (Maxent; [23], [24]), which is currently regarded as one of the best methods available [25]. Output from Maxent models is the probability of occurrence for a given landuse type, which we tentatively term “suitability” throughout this paper (see Discussion). We used response curves to describe the (partial) effects of the most important variables in the models. Responses are selected for best predictive ability [see 24 for details]. Although they may sometimes elucidate functional relationships, it is important to point out that they do not represent any proof of relationships in a null hypothesis testing framework. The area under the receiver-operating characteristic (AUC) from cross-validation was used as a standard measure of model quality. We included details on modelling and model evaluation in File S1. Table 1 gives an overview to environmental variables used for each of the four models.

**Table 1:** Environmental variables used for Maxent models.

Variable	Type	AGR	ANIM	PAST	HG
Altitude	CON	X	X	**X**	X
Annual temperature range	CON	**X**	X	X	**X**
Annual temperature maximum	CON	X	X		
Maximum temperature of warmest month	CON	X			
Mean monthly temperature range	CON	X	X	X	X
Mean annual temperature	CON	X		X	X
Mean temperature of the coldest quarter	CON	X	X	X	X
Mean temperature of the warmest quarter	CON	X	X	X	X
Mean temperature of the wettest quarter	CON	X	X	X	X
Mean temperature of the driest quarter	CON	X	X	X	X
Annual temperature minimum	CON	X	X	X	
Minimum temperature of the coldest month	CON	X	**X**		X
Annual precipitation	CON	**X**	X	X	**X**
Precipitation of the wettest quarter	CON	**X**	**X**	X	
Precipitation of the driest quarter	CON	X	X	X	X
Precipitation of the warmest quarter	CON	X	X	**X**	**X**
Precipitation of the coldest quarter	CON	X	X	X	
Precipitation seasonality	CON	X	X	X	X
Soil	CAT	X	**X**	**X**	**X**

CON denotes continuous, CAT categorical variables used for Maxent modelling of agriculture (AGR), sedentary animal husbandry (ANIM), nomadic pastoralism (PAST) and hunting-and-gathering (HG). Variables used for a particular landuse model are denoted by X, whereas red printing indicates that a variable was often among the three most influential variables (based on 10 model runs). Restriction to chosen variables was based on preliminary model trails (not detailed here) and some logical reasoning (e.g., using climate variables in relation to season rather than to month, considering the trans-equatorial extent of our study). See main text for the shape of effects of some variables in the models. Note that Maxent ENM is not designed as null hypothesis test on the effect of particular variables.

We used a FAO landuse classification (http://www.fao.org/nr/lada/index.php?/maps/Maps-lada/) as an independent assessment our model predictions. We converted modelled suitability into a presence-absence prediction on landuse types (see File S1 for details). FAO categories were re-classified to fit our landuse types, but only for agriculture we see enough reliability to present in detail data of a comparison between FAO “reality” and our model prediction. We must point out that uncertainty was encountered in how to classify various “mixed” categories in the FAO data, and we applied an “agriculture if in doubt” strategy. Therefore, results based on the FAO data must be viewed with care.

### Further analyses

To map areas where two landuse types (*lut*1 and *lut*2) would have similar, and high, values of suitability, we designed an ‘index of shared suitability’ (ISS).
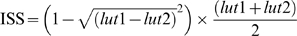
ISS will reach values close to one where two landuse types are both well suited; it will be close to zero where one is clearly better than the other, or where both are bad. We presume that high ISS are a precondition of conflicts over the “best” form of landuse, which may translate into conflicts between cultures with different traditions in this respect.

We used population data provided by LandScan (for the year 2005; http://gcmd.nasa.gov/records/GCMD_Landscan.html) to relate agricultural suitability to population density (data per km^2^, interpolation averages for our ca. 5×5 km cells). We extracted a sample of 2000 randomly chosen points (using Hawth's tools, http://www.spatialecology.com/htools/) and carried out regression analyses on this sample, using a log_10_(x+1) transformation of population data (software Statistica 8). We assessed the statistical significance of linear relationships by using spatial correlation (adjusted degrees of freedom, Dutilleul's method, implemented in software by [26]) to account for spatial non-independence in raster data. We calculated both, a linear regression of suitability and log-transformed population density, and a breakpoint regression. We extrapolated these models to the entire region, re-exponentiated data, and mapped residuals from the “true” population data.

Following the approaches of [8] and [10] to test Diamond's hypothesis [1] on why some regions are more “powerful” politically and economically than others, we also related modelled suitability for agriculture to economic data. Unlike earlier authors we used a correction of gross domestic product data (GDP) known as purchasing power parity (PPP; e.g. [27]). PPP acknowledges that currency exchange rates sometimes do not reflect the true value of wealth generation due to trade imbalances and/or active manipulation of exchange rates. One PPP currency unit may be interpreted as having the same purchasing power of a locally produced good. We used data of GDP-PPP per grid cell for the year 2000 ([28]; available at http://gecon.yale.edu/, version 2.11).We applied the same analytical procedures as for agricultural suitability - population density correlations.

We used a database of post-1945 armed conflict (Peace Research Institute, Oslo (PRIO): georeferenced localities and approximate radius of conflict; http://www.prio.no/) for an initial quantitative evaluation of ISS (agriculture–pastoralist) predictions. Sites were classified as “conflict” or “no conflict”, and a logistic regression model with ISS as predictor was applied to a random sample of 500 localities. We controlled for spatial autocorrelation by including spatial eigenvectors as predictors [29, selected according to default settings in software by 26].

## Results

### 1) Model predictions

**Figure 1:**
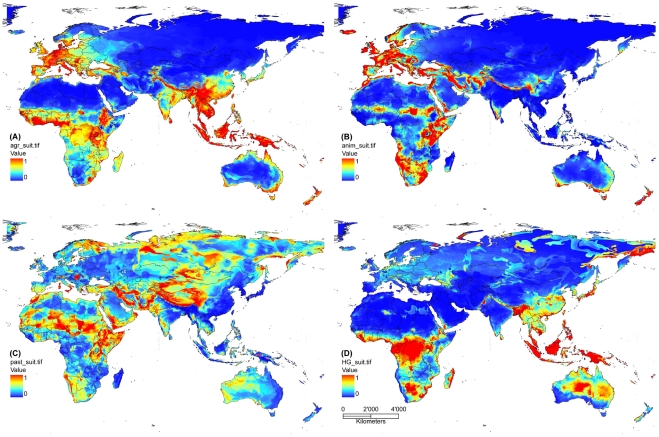
Modelled “suitability” (probability of occurrence, Maxent) for (A) agriculture, (B) sedentary animal husbandry, (C) nomadic pastoralism, and (D) hunting and gathering.

Model output for the four landuse types as presented in Fig. 1, GIS-compatible data are available for detailed inspection and further use (Data Sets S1, S2, S3, S4).

#### Agriculture

Suitability for agriculture is clearly non-randomly distributed, with regions of high values being found in the wet tropics and in some temperate regions, particularly Western Europe, in the Ethiopian highlands, East Africa around Lake Victoria, subtropical East Asia, the Himalaya foothills, south-eastern Australia and New Zealand. Many fine-scaled patterns cannot be discussed here in detail for space limitation.

With an AUC of 0.870 (average of 10 models), predictive qualities of the model can be considered good. The most influential environmental predictor variables (from their relative contribution to 10 model runs) are detailed in Table 1. These three variables explained, on average, >60% of data variance.

Response curves (after consideration of the effects of other variables in the model) give account of how variables entered ENMs for best predictive performance; they do not represent tests of a priori hypotheses. For agriculture, they indicated a generally positive relationship between agricultural suitability and annual precipitation, a negative link with annual temperature range, and a unimodal relationship with precipitation in the wettest quarter (with maximum suitability in regions of relatively low precipitation). Notably, climate appeared much more important than soil types in the multivariate model.

#### Sedentary animal husbandry

Similar to agriculture, western, central and southern Europe, East Africa, Iran, southern Australian and New Zealand are considered suitable for sedentary animal husbandry. In contrast to agriculture, however, tropical West Africa and tropical Asia are considered highly unsuitable, whereas southern Africa is indicated as a suitable region.

Model quality is good with an AUC of 0.899. The most important variables are detailed in Table 1. Suitability of animal husbandry is very low at minimum temperatures below −15°C, whereas it follows a unimodal function with a peak at ca. 5°C above that. Gleyosols, planosols, andosols, cambisols and luvisols were among the soil types associated with high suitability for animal husbandry in the model. With regard to precipitation of the wettest quarter, suitability had a sharp unimodal peak at low precipitation and dropped to zero above that.

#### Nomadic pastoralists

The predicted suitability of nomadic pastoralist follows a different pattern than that of agriculture, with good areas in the southern Sahara, the drier parts of East Africa, parts of the Arabian peninsula, Iran, arctic Scandinavia, central Asia and parts of Siberia. Parts of the Mediterranean and eastern Hungary are also suitable.

However, the model was considered markedly less precise with an average AUC of 0.683. Soil type was very important for predicting this trait, explaining 47% of variance on average. Histosols, gleyosols, leptosols and phaeocems were soil types that indicated the occurrence of nomadic pastoralists in the model. The three most important variables (Table 1) explained, on average, almost 70% of variance. Suitability decreased steeply with precipitation in the warmest quarter, and it increased monotonically with altitude (partial effects in the multivariate model).

#### Hunters-and-Gatherers

Hunters-and-gatherers find highly suitable habitat, according to our model, in the wet tropics of Africa and the Malay Archipelago, in the deserts of southern Africa and Australia, and parts of the Arctic and Subarctic, particularly eastern Siberia. Interestingly, highly suitable areas are also located around the lower Ganges and Brahmaputra rivers (i.e., Bangladesh and eastern India), the Mekong delta, and across large parts of Japan. The classification of fishing as parts of a hunter-and-gatherer strategy may be responsible for this effect.

The model is considered good with an AUC of 0.867. The three most important variables (Table 1) explained an average of 69% of data variability. Modelled suitability increased with increasing precipitation, declined with increasing annual temperature range, and was typically high at soil types such as cryic leptosol, gleyosol, andosol, ferralsol or acrisol.

#### Comparison to FAO landuse data

**Figure 2:**
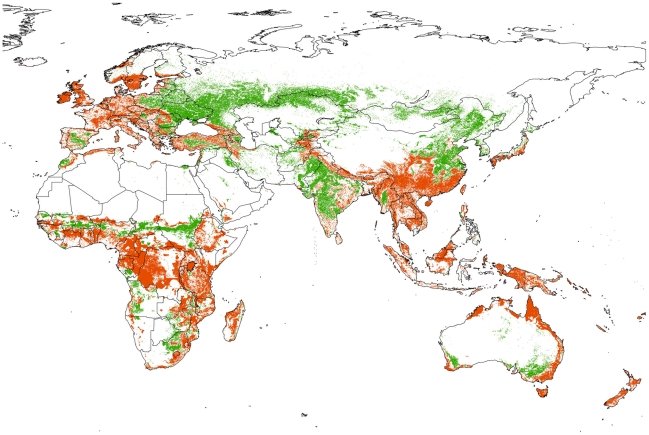
Deviations between Maxent model prediction for the occurrence of agriculture and a reclassified FAO landuse map. Green areas denote regions where FAO indicates agriculture, but the model does not predict it; red areas highlight where the model predicts agriculture, but FAO does not record any. In white areas, model predictions and FAO maps agree. Note that apparently solid regions of deviation often show fine-scale pattern at higher resolution. See Methods for caveats in the reclassification of FAO landuse data.

Our model for the presence of agriculture (threshold value of 0.18, applied to cumulative Maxent output) leads to mispredictions in certain parts of the world (Fig. 2) that require further investigation – for example, we predicted the absence of agriculture from large farming areas of eastern Europe, southern Siberia, northern China, parts of India, and the Sahel zone south of the Sahara. On the other side, we predict agriculture in regions where it seems less widespread according to FAO – e.g. central Europe (probably the effect of centuries of reforestation efforts [30]), wet-tropical Africa, southern China and Southeast-Asia, New Guinea, the Australian east coast and New Zealand.

For animal keeping (combined for nomadic or sedentary types, as FAO data is unspecific; not shown), potentially important deviations between our predictions (threshold values of 0.13 and 0.09, respectively, applied to cumulative output) and FAO's equivalent data are the factual presence of livestock in many drier part of Australia, western Madagascar, southern China, parts of the Arabian deserts, and much of Kazakhstan.

### 2) Shared suitability

**Figure 3:**
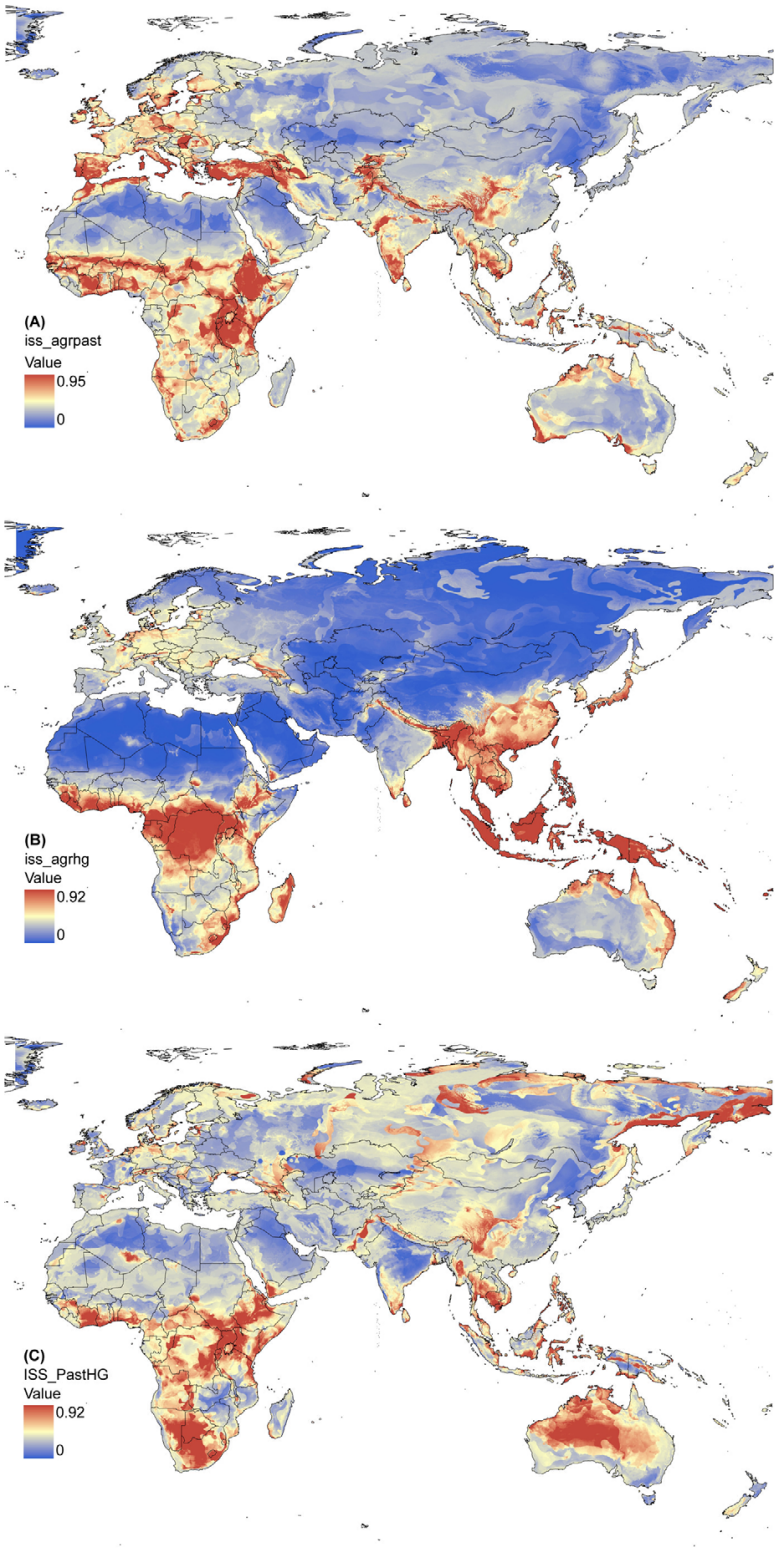
Index of shared suitability (ISS) for (A) agriculture vs. nomadic pastoralism, (B) agriculture vs. hunting and gathering, (C) nomadic pastoralism vs. hunting and gathering. High ISS values indicate that a land is of high suitability for both landuse forms.

Maps of ISS for some landuse pairs yielded highly interesting results (Fig. 3), especially when it is considered that they are based on just soil and climate parameters. High ISS for agriculture vs. nomadic pastoralism, in particular, occurred in many regions known for violent conflicts over land and resources. Some of those are even ethnically associated with agriculturalist vs. nomad – conflicts (e.g., in Rwanda, Burundi, Congo's Kivu region, Dafur, southern Mali, parts of Ethiopia), whereas this seems difficult currently to reconcile for regions such as northern Ireland, “Kurdistan”, northern Israel and Lebanon, northern Ivory Coast and parts of central Asia. This does not rule out, however, that such landuse conflicts lay once at the heart of some of these often long lasting struggles.

A quantitative test of ISS prediction on post-1945 conflict data (see Methods) indicated that ISS for agriculture vs. nomadic pastoralism made a significant contribution to predict conflict occurrence from a logistic regression model (containing ISS and two spatial eigenvectors as predictors; N = 500, χ^2^ = 157.5, p<0.0001; partial effect of ISS: t = 2.58, p = 0.01). Predictive accuracy was ca. 68%. However, it has to be recognized that the conflict database did not allow a detailed distinction of which conflicts were related to landuse. The observed effect is therefore encouraging to our hypothesis, but more meaningful tests will require a careful classification of conflict causes that are well beyond the scope of this paper.

Similarly suggestive, ISS for agriculture vs. hunter-and-gatherers was high in the Congo region, coastal West Africa, and the Asian tropics and subtropics. Incidentally, this largely overlaps two of Diamond's prime examples of the genocide on hunters-and-gatherers, the expansion of the Bantu and of the Austronesians [1]. High ISS for pastoralist vs. hunter-and-gatherers identified large areas in southern and eastern Africa (reminiscent of the scattered occurrences of Khoisan ethnic groups), and in much of the drier part of Australia (where pseudo-nomadic cattle and sheep herding is carried out on formerly aboriginal land).

### 3) Agriculture and population density

**Figure 4:**
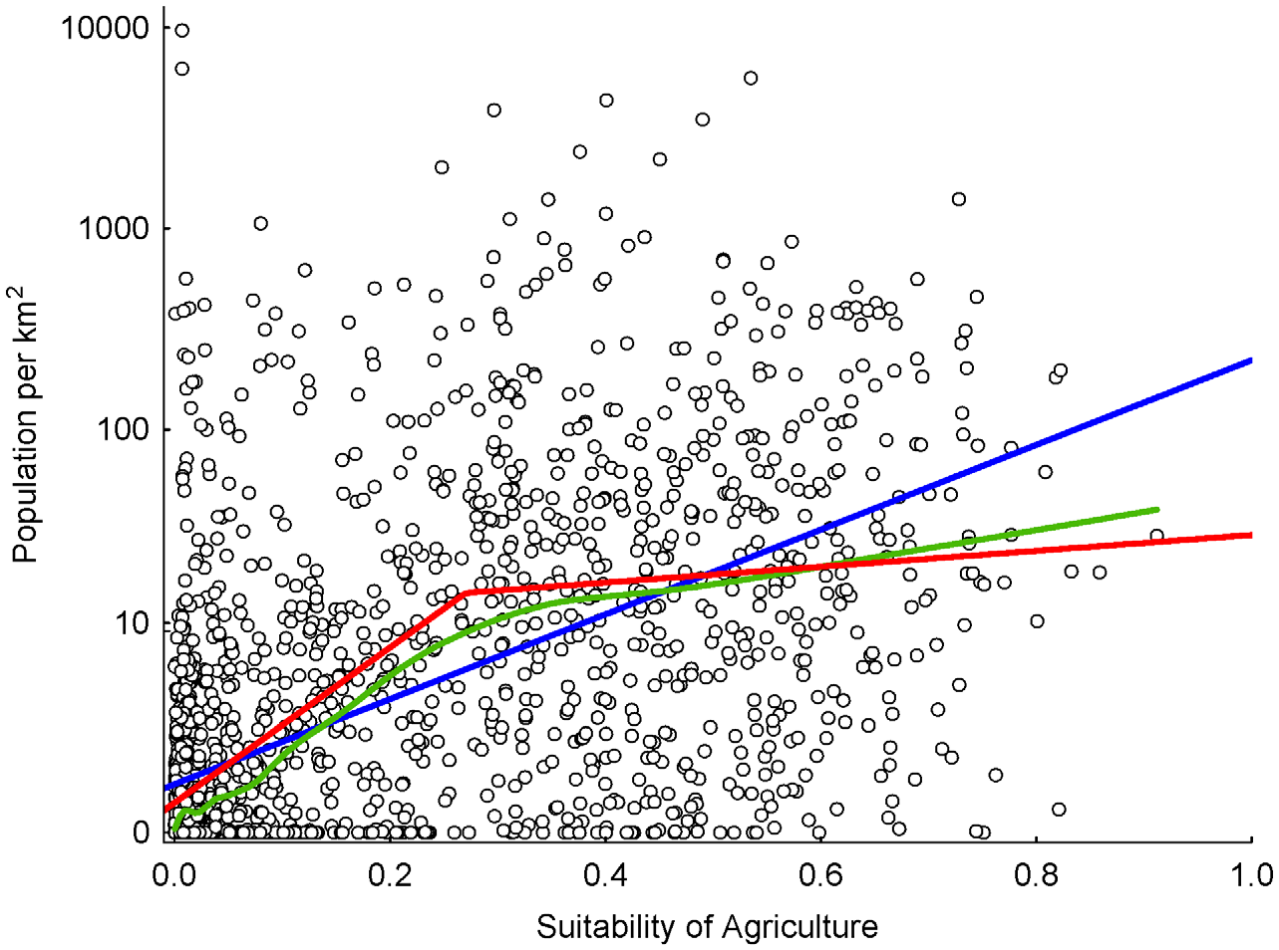
Suitability for agriculture and population density in 2000 randomly chosen grid cells. The green line represents a locally weighted regression, a largely data-driven representation of the relationship. The blue line is a linear regression (on log-transformed population data; 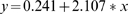 (r^2^ = 0.338; two parameters estimated). The red line is a breakpoint regression 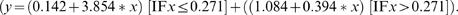 The breakpoint model (r^2^ = 0.380) required estimating four parameters. Note that regression parameters refer to log_10_(Population Density+1) (see Methods), whereas y-axis values were re-exponentiated for display.

Suitability for agriculture is significantly related to log-transformed population density (N = 2000; adjusted for spatial non-independence: F_adj_ = 26.6, df_adj_ = 52.0, p<0.001) and explains 33.8% of data variability. However, a closer look at the data indicates that the relationship is not linear (Fig. 4). Rather, agriculture is strongly related to (log-transformed) population density below a value of ca. 0.3, whereas there is only a weak relationship above this. A breakpoint regression accounts for this, and explained variance increases to 38.0%. An alternative linear regression on non-transformed data, using a negative binomial error structure, did not lead to better results.

**Figure 5:**
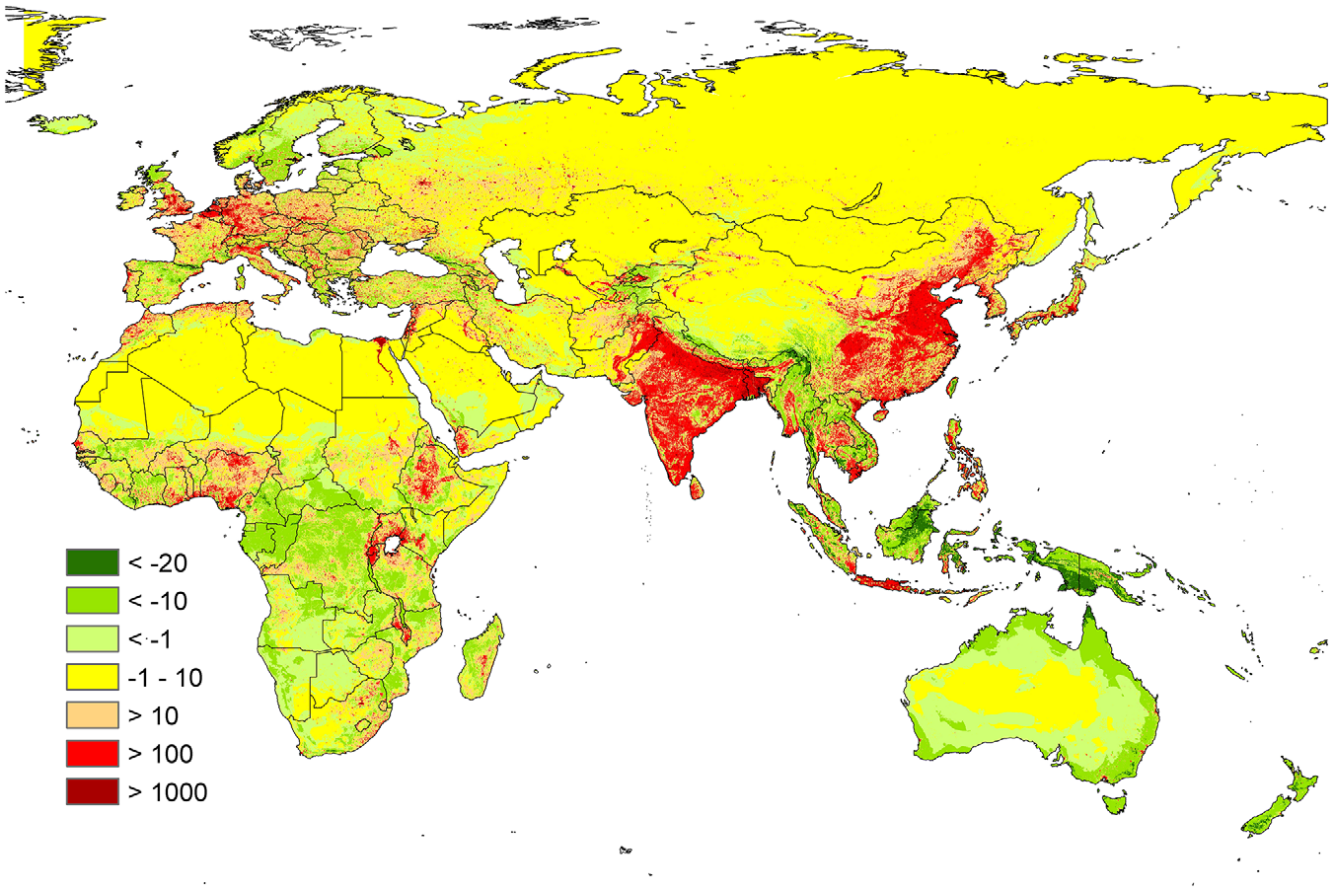
Residuals of population densities (people per km^2^) from expected values based on the breakpoint regression reported in Fig. 4. Yellow regions indicate good fit between population data and our model predictions, green region highlight areas where the model overpredicted population densities (less people found than predicted), red regions indicate that more people are present than expected by the model. Note that the asymmetric distribution of error (larger positive than negative residuals) is a consequence of modelling on log-transformed data and does not require a functional interpretation.

We present here only a map of residuals (Fig. 5) from the (log−) breakpoint model, but differences to the (log−) linear regression are minor. There are clear patterns of positive and negative deviations of population density from those values predicted by our modelled suitability for agriculture. Notably, as indicated in Fig. 4, these deviations are more common above the breakpoint value, i.e. in regions of generally high suitability of agriculture (Fig. 1A), while the model is better in predicting where only few people should live.

We found only much weaker correlations between log-transformed population density and suitability for other landuse types (spatial correlations: sedentary animal husbandry, r^2^ = 0.070, F_adj_ = 6.1, df_adj_ = 80.4, p = 0.016; nomadic pastoralism, r^2^ = 0.023, F_adj_ = 5.4, df_adj_ = 230.3, p = 0.021; hunting-and-gathering, r^2^ = 0.037, F_adj_ = 3.6, df_adj_ = 93.8, p = 0.062). We also evaluated multivariate models of linear combinations of the four landuse suitabilities, using AIC-based model selection [31]. A model containing the suitability of all three landuse types (all except animal husbandry) as predictors (r^2^ = 0.357) is judged better than the univariate linear model presented in Fig. 4 (ΔAIC>57, not corrected for spatial effects). However, partial effects of the suitability for hunting-and-gathering and nomadic pastoralism would be negative, making no intuitive sense from a causal perspective. Similar effects were observed in a generalized additive model (GAM, allowing non-linearity in the shape of links). GAM accounted for ca. 41% of data variability, which is not much better than the univariate breakpoint model (Fig. 4). We conclude, from these and further modelling attempts not reported here, that suitability for agriculture is by far the most important landuse type to predict population density, but that remaining variability is largely caused by factors other than landuse type (in our broad categorization).

We found a weaker, yet significant linear relationship between suitability for agriculture and 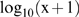-transformed cell-wide GDP-PPP (N = 2000, r^2^ = 0.153, F_adj_ = 11.0, df_adj_ = 60.7, p = 0.002), while a breakpoint regression (steep positive relationship to a suitability of 0.1, weaker positive relationship above that; data not shown) explains only slightly more data variability (r^2^ = 16.4).

Correlations with agricultural suitability are not dependent on the inclusion of zero values (i.e., cells without inhabitants or GDP; excluding these would led to p<0.001, respectively p<0.01, in spatial correlations). However, they were retained in the data presented because they may be of functional significance, and because exclusion of zeros can lead to bias in spatial data [32].

## Discussion

### Climate, soil and their consequences

We found a high correspondence of landuse with climate and soil parameters, evidenced by our ability to predict in some detail the spatial distribution of the former from the latter. We conclude that the distribution of these basic geographical factors had an important effect on the spread of landuse types, and therefore on cultural traits associated with them. Furthermore, our data indicated that one landuse type, agriculture, is related to population density and even economic strength of regions, explaining statistically 38%, respectively 16%, of data variability of current conditions. Fig. 4 indicate that this, in particularly, explains low populations densities of regions highly unsuitable for agriculture.

Furthermore, deviations from model predictions are highly non-randomly distributed geographically (Figs. 2 & 5). Part of our aim was to also explore where more complex factors besides climate and soil might be necessary to explaining landuse strategies. Some deviations are readily explicable by our simplifying assumptions, such as not considering local-scale, rural-to-urban food transport or water availability from rivers. These aspects are of lesser interest here, but some examples are reported in File S2.

There are also less trivial deviations that deserve further study. For example, contrary to the observed, our model indicated high suitability for agriculture, hence high population density in some tropical regions, while there are more people than predicted in parts of Europe, India and China. Such deviations invite ad-hoc hypotheses and speculations that may be investigated in further research (see File S2 for details). For example, agricultural efficiency may differ between regions; cultural conservatism (non-acceptance of more efficient forms of landuse) or disease may have inhibited population growth [e.g. 33, p. 233]. “Overpopulated” regions (according to model predictions; red in Fig. 5) could also indicate unsustainably high numbers of people, while “under-populated” places (green in Fig. 5) may point out wilderness areas that should be given conservation priority [34] because they may be predicted to “fill up” in the future. More thorough analyses of what explanation applies where, in combination with applying our model to climate change scenarios, may provide useful data for global policy planning.

Our measure of “suitability” (precisely, probability of occurrence; see Methods) is not equivalent to productivity, but we assume it to be largely monotonically related to it. More sophisticated models have been used to assess agricultural yield in the context of predicting climate change effect [e.g., 35], but we nevertheless see a value in our independently derived data that may well be used for similar tasks in further research on this topic of applied relevance [36]–[38].

### Predicting conflicts

Overlaps of suitability between various pairs of landuse types coincided with many actual conflict regions (see Results), implying that climate and soil could be, at least indirectly, related to the occurrence such conflicts. Recently, other researchers also suggested links between climate, violent conflict and human economy [39], [40].

Shared suitability does not necessarily imply violent landuse conflicts, however. High ISS may simply lead to mixed strategies in landuse practices (see File S2 for examples). It seems likely that the key ingredient leading to either adopting mixed landuse strategies or violent conflict is ethnicity. This hypothesis would need to be investigated in the future. If different landuse traditions coincide with other differences between peoples (e.g. language, physical appearance, religion), not only violence may be more likely but cultural self-definition may also prevent people from adopting some of “the others'” landuse techniques despite potential economic benefits (cf. Vikings in Greenland, [5]). We suggest that intersecting our ISS maps with regions near ethnic, religious or linguistic boundaries may lead to further refinement in predicting potential conflict regions.

### Ecological niche models of landuse traits: properties and caveats

Several properties of ecological niche modelling in its usual application (i.e., predicting species' geographical ranges) are critically assessed, and some of these problems also apply here. Species are assumed to be unrestricted in dispersal to all suitable sites [but see 41], [42]. In our context, we find it realistic to assume that all peoples have knowledge of all four types of landuse. More important, species' niches are assumed to be constant throughout their range (i.e., there is no local adaptation). Possibly because of this often unrealistic presumption, geographic distributions of wide-ranging species are more difficult to predict than geographically restricted ones [43]. Our application on landuse traits certainly contains such local adaptation due to peoples' choice of utilized organisms. Growing rice will need other climate conditions than growing wheat, while herding reindeer will require other conditions than herding camels. Furthermore, all four landuse traits are globally spread. In light of this, it is surprising that our models generally are of good predictive quality as judged by AUC.

Model predictions are based on present day occurrences of landuse types, and hence the effects of competition with other landuses [cf. 44]. As a consequence, modelled effects may not always point out best regions per se, but rather those where a landuse type is a sufficiently good competitor to other practices. Nomadic pastoralism, for example, may not be better on soils poor in nutrients, but the absence of agriculturalists from bad soils may pose a crucial advantage.

We noticed throughout our results that sharp changes in mapped values sometimes coincided with country borders or other culturally defined boundaries (see File S2 for examples). Possibly, courses of history were sometimes subtly affected by climate and soil and their consequent effects on the quality of lands (e.g., worthiness of defending). Some coincidences of this type may be expected by chance. Deciding with certainty whether this is a significant pattern would require thorough study beyond the scope of this paper.

### The value of a simple model

Our simplistic exercise showed that a “geo-deterministic” approach can predict surprisingly many features of human cultural geography without any explicit cultural or historical assumptions. Although many deviations require further factors for a satisfying explanation, the nature of these deviations invite the generation of hypotheses for further research. Our ‘null model’ offers a highly parsimonious, empirically supported explanation to the question of why some regions are more “powerful” than others, supplementing the idea of a historical effect operating through the timing of transition to agriculture [2].

In some instances, our model may actually provide a simpler explanation. Putterman [10], for example, suggested that the dominance of Western European cultures indicates the transmission of “civilization” traits (other than knowledge on agriculture) from regions of first domestication. Our data indicate that higher climatic suitability may have been sufficient for Europe to “catch up”, allowing for much higher population densities than, e.g., the “fertile crescent” region. Models of “suitability” under past climatic scenarios may be helpful to evaluate this. Similarly, models applied to predicted future climatic scenarios may be useful to anticipate changes of the economic suitability of landuse types.

However, our model has clear deviations in some regions that may well be explicable by the availability of animals and plants suitable for domestication (e.g., central Africa). Apart from that, and more importantly, we know that human societies and economies went through historical development, so ignoring history may not always be the best strategy to understand causalities. This problem occurs also with other research questions in biogeography, e.g. when investigating global biodiversity patterns [45]–[47]. Nevertheless, our ‘null model’ will be a useful tool in identifying regions that require further investigation to understand additional processes that shape the distribution and performance of human economic traits.

## Supporting Information

Table S1 Georeferenced presence records of four landuse types used for modelling.
(0.06 MB XLS)

File S1 Methods details: ecological niche modelling and validation.
(0.03 MB DOC)

File S2 Details and interpretation of some patterns.
(0.04 MB DOC)

Data Set S1 Maxent model output: agriculture (ASCII grid).
(21.38 MB ZIP)

Data Set S2 Maxent model output: sedentary animal husbandry (ASCII grid).
(22.30 MB ZIP)

Data Set S3 Maxent model output: nomadic pastoralism (ASCII grid).
(20.86 MB ZIP)

Data Set S4 Maxent model output: hunters-and-gatherers (ASCII grid).
(21.60 MB ZIP)
