# Supplementary material for: Is the Spatial Distribution of Mankind's Most Basic Economic Traits Determined by Climate and Soil Alone?
Source: PLoS One. 2010 May 5;5(5):e10416. doi: 10.1371/journal.pone.0010416 (PMC2864741; doi:10.1371/journal.pone.0010416)
Supplement: File S1 — Methods details: ecological niche modelling and validation. (0.03 MB DOC) [file pone.0010416.s002.doc]

**S2: Methods details: Ecological niche modelling and validation**

**1) Ecological niche modelling**

Despite a large number of different methods being available, and continuous further development, the basic idea can be described as a three-stage process.

1. Data on known presences, more rarely also on confirmed absences, are used in combination with data on environmental conditions (most often climate parameters) to construct a statistical model of the ecological niche (Soberon 2008) of a species. The model specifies under what set of environmental conditions a species is likely to be found (implying that it survives, grows and reproduces there).
2. With environmental variables being available as a raster grid over large areas, the model can be extrapolated to map the suitability of a landscape for a given species (implying some critical assumptions discussed below). Model output is, in appropriate transformation, a measure of “probability of occurrence” (under the basic assumptions of niche modelling, see Discussion).
3. If a clear presence-absence prediction is required (which depends on the research question), a threshold value is applied that allows reclassifying the ‘suitability’ map to binary data (‘present’ or ‘absent’). A suitability of 0.5 (on a 0-1 scale) has often been applied, but Liu et al. (2005) and Jiminez-Valverde & Lobo (2007) have shown that case-specific approaches of choosing thresholds lead to better predictions. For comparison with FAO landuse classification (see main text), we applied the method of finding a threshold where the receiver-operating characteristic (ROC) is in its upper left corner of the graph (i.e., where commission and omission errors are both minimized), as advised by Liu et al. (2005).

A large variety of methods are currently used to provide ecological niche models. Maximum Entropy modelling (Maxent; Phillips et al. 2006, Phillips & Dudik 2008) is considered one of the best methods available (Elith et al. 2006). We used Maxent to predict the “ecological niches” of land use traits based on climate and soil variables, using a selection of potentially useful variable used for models (main text, Table 1). Due to the properties of Maxent, overfitting of models by using too many independent variables (a common problem in many multivariate modelling approaches) is not of concern (Phillips & Dudik 2008). We used default settings and a logistic output format (except where noted otherwise), which can be interpreted as probability of occurrence. We call it “suitability” in our context. Maxent is designed to be used with non-systematically collected “presence-only” records. Model quality is robust down to relatively small sample sizes (≥ 10 records; Wisz et al. 2008).

**2) Model evaluation**

We used cross validation, a standard method, to assess the predictive qualities of the models. Models are initially fitted to a randomly chosen subset of the data (in our case, 70 percent of records; “training data”). The remaining, 30 percent “test data” are then used to assess the prediction in the light of these independent data. Using different randomized test and training data, this procedure was repeated ten times for each model, and average quality assessments are reported. Model quality for “test” data (i.e., external model validation) was measured by the area under the receiver-operating characteristic curve (AUC), a metric that remains standard in model evaluation despite some undesired properties (Lobo et al. 2008, Peterson et al. 2008). AUC increase from 0.5 to 1 as one moves from a random prediction to a perfect model. The advantage in AUC lies in the possibility of assessing model quality across the whole range of possible threshold values (see above), and hence independent of this secondary decision.

However, for a final model (used for map and further investigation) we used all data available to assure maximum quality of the model (assuming that more data leads to better models; Wisz et al. 2008).

**References**

Elith J, Graham CH, Anderson RP, Dudik M, Ferrier S, Guisan A, Hijmans RJ, Huettmann F, Leathwick JR, Lehmann A, Li J, Lohmann LG, Loiselle BA, Manion G, Moritz C, Nakamura M, Nakazawa Y, Overton JMcC, Peterson AT, Phillips SJ, Richardson K, Scachetti-Pereira R, Schapire RE, Soberon J, Williams S, Wisz MS, Zimmermann NE (2006) Novel methods improve prediction of species’ distributions from occurrence data. Ecography 29: 129-151.

Jiminez-Valverde A, Lobo JM (2007) Threshold criteria for conversion of probability of species presence to either–or presence–absence. Acta Oecologia 31: 361–369.

Liu C, Berry PM, Dawson TP, Pearson RG (2005) Selecting thresholds of occurrence in the prediction of species distributions. Ecography 28: 385-393.

Lobo JM, Jiminez-Valverde A, Real R (2008) AUC: a misleading measure of the performance of predictive distribution models. Global Ecology and Biogeography 17: 145–151.

Peterson AT, Papes M, Soberon J (2008) Rethinking receiver operating characteristic analysis applications in ecological niche modelling. Ecological Modelling 213: 63–72.

Phillips SJ, Anderson RP, Schapire RE (2006) Maximum entropy modeling of species geographic distributions. Ecological Modelling 190: 231–259.

Phillips SJ, Dudik M (2008) Modeling of species distributions with Maxent: new extensions and a comprehensive evaluation. Ecography 31: 161-175.

Soberon J (2008) Grinellian and Eltonian niches and geographic distribution of species. Ecology Letters 10: 1115 – 1123.

Wisz MS, Hijmans RJ, Li J., Peterson AT, Graham CH, Guisan A, et al. (2008) Effects of sample size on the performance of species distribution models. Diversity and Distributions 14: 763–773.
