# Supplementary material for: Is the Spatial Distribution of Mankind's Most Basic Economic Traits Determined by Climate and Soil Alone?
Source: PLoS One. 2010 May 5;5(5):e10416. doi: 10.1371/journal.pone.0010416 (PMC2864741; doi:10.1371/journal.pone.0010416)
Supplement: File S2 — Details and interpretation of some patterns. (0.04 MB DOC) [file pone.0010416.s003.doc]

**S3: Details and interpretation of some patterns**

**1) Deviations of models and reality as consequences of model simplicity**

Local-scale food transport from rural to urban areas feeds city people, but our model maps all urban agglomerations as positive deviations from model expectations (main text, Fig 5).

Some larger-scale patterns of underestimation of agriculture or population density, for example in southern Siberia (linked to the Transsiberian Railway), can probably also be explained in this way.

We did not account for water availability other than by precipitation, therefore we heavily underestimate the potential for agriculture and hence population density in some river valleys in dry regions, such as along the Egyptian Nile, or between Euphrates and Tigris in Iraq.

Large-scale overestimates of population density in Australia and New Zealand are corroborated by the fact that these countries are major net exporters of foods.

We observed an underestimate of agriculture in part of Eastern Europe (main text, Fig. 3), particularly in Ukraine, the “wheat basket” of the former Soviet Union – but predictions of population densities in the region are quite good (main text, Fig. 5), indicating that agricultural productivity may be indeed much lower then further west.

**2) Some deviations requiring more complex explanations, and suggestions thereof**

Our model indicated high suitability for agriculture, hence high population density, on the large tropical islands Borneo and New Guinea, as well as in parts of the Congo basin. On the other hand, there are more people than predicted in parts of Europe, India, China, on the Indonesian island of Java and in parts of Africa.

On the one side, agricultural efficiency may differ between regions due to neglected environmental factors (e.g. variation in yield between crops planted) or due to techniques used (e.g. variation in intensity due to fertilization, plough depth, traditional irrigation systems, weed control, etc.;). Largest under-predictions of populations sizes (dark green, Fig. 5) occurred in central-eastern Borneo, southern New Guinea, and Australia’s Cape York, where many people are still hunter-and-gatherers (indicating a historical effect). Disease prevalence has been discussed as a factor inhibiting population growth in tropical Africa (Reader 1997, pp. 233).

On the other side, “overpopulated” regions (according to model predictions) could actually indicate unsustainably high numbers of people. Dependency of some of the red-flagged regions in Fig. 5 (e.g., Rwanda, Burkina Faso) on food imports may be supporting this. A more thorough analysis of where this explanation applies, in combination with applying our model to climate change scenarios, may provide useful data for policy planning.

Over-predicted regions (green in Fig. 5) may point out under-populated wilderness areas that could be expected to be “filled up” in a matter of time, and should hence be given some conservation priority (Mittermeier et al. 1998). The current spread of oil palm plantations throughout low-population rainforest regions of Southeast Asia (e.g. Fitzherbert et al 2008, Koh & Wilcove 2008) may be interpreted as the beginning of such a process, driven by a new (i.e., African) crop plant and a novel global economic demand for the product.

**3) Non-violent consequences of high ISS: Examples of mixed strategies.**

Many farmers across Europe are also keeping (sedentary) livestock (high ISS between these landuse types across Europe, not shown in detail).

Farmers in the Swiss Alps send their cattle, sheep or goats to seasonal high-altitude pastures (the “Alp”, a nomadic strategy).

Farmers in northern Borneo, in the New Guinea highlands or in northern Ivory Coast get a significant proportion of their protein from hunting (personal communications, but see also manifold literature on African “bushmeat”).

**4) Coincidence of sharp changes in modelled values with political region: some examples**

Western borders of Poland in Fig. 1 (A, B).

Borders of Rwanda, Burundi and Uzbekistan in Fig. 5

Bretagne (France; the only celtic linguistic region in mainland Europe), Cornwall (Great Britain, with occasional independence movements), Northern Ireland (Fig. 3A).

Lesotho (Fig. 1A, C)

**References**

Fitzherbert EB, Struebig MJ, Morel A, Danielsen F, Brühl CA, Donald PF, Phalan B (2008) How will oil palm expansion affect biodiversity? [Trends in Ecology & Evolution](http://www.sciencedirect.com/science/journal/01695347) 23: 538-545.

Koh LP, Wilcove DS (2008) Is oil palm agriculture really destroying tropical biodiversity? Conservation Letters 1: 60-64.

Mittermeier RA, Myers N, Thomsen JB, da Fonseca GAB, Olivier S (1998) Biodiversity hotspots and major tropical wilderness areas: Approaches to setting conservation priorities. Conservation Biology 12: 516-520.

Reader J (1998) Africa. Biography of a Continent. London: Penguin Books.
